# The HOX Gene Family’s Role as Prognostic and Diagnostic Biomarkers in Hematological and Solid Tumors

**DOI:** 10.3390/cancers17020262

**Published:** 2025-01-15

**Authors:** Kaci Kopec, Danielle Quaranto, Nicole R. DeSouza, Tara Jarboe, Humayun K. Islam, Augustine Moscatello, Xiu-Min Li, Jan Geliebter, Raj K. Tiwari

**Affiliations:** 1 Department of Pathology, Microbiology and Immunology, New York Medical College, Valhalla, NY 10595, USA; kkopec@student.touro.edu (K.K.); dquarant@student.touro.edu (D.Q.); ndesouza@student.touro.edu (N.R.D.); tseymour@student.touro.edu (T.J.); humayun.islam@wmchealth.org (H.K.I.); a_moscatello@nymc.edu (A.M.); xiumin_li@nymc.edu (X.-M.L.); raj_tiwari@nymc.edu (R.K.T.); 2 Department of Otolaryngology, New York Medical College, Valhalla, NY 10595, USA; 3 Department of Dermatology, New York Medical College, Valhalla, NY 10595, USA

**Keywords:** homeobox (HOX) genes, biomarker, cancer

## Abstract

The HOX gene family encodes for regulatory transcription factors that play a crucial role in embryogenesis and differentiation of adult cells. This highly conserved family of genes consists of thirty-nine genes in humans that are located in four clusters, A–D, on different chromosomes. While early studies on the HOX gene family have been focused on embryonic development and its related disorders, research has shifted to examine aberrant expression of HOX genes and the subsequent implication in cancer prediction and progression. Due to their role of encoding master regulatory transcription factors, the abnormal expression of HOX genes has been shown to affect all stages of tumorigenesis and metastasis. This review highlights the novel role of the HOX family’s clinical relevance as both prognostic and diagnostic biomarkers in hematological and solid tumors.

## Simple Summary

Investigation of cancer biomarkers for prognostic, diagnostic, and therapeutic applications has enhanced patient outcomes in hematological and solid cancer types. Cancer biomarkers are discovered by evaluating genetic dysregulation compared to normal samples. Homeobox genes, genes that are key players during embryonic development, have been shown to have dysregulated expression patterns in multiple cancer types. Therefore, this review covers the dysregulation of HOX genes and their encoded factors and the HOX family’s role as potential cancer biomarkers.

## 1. Introduction

**Figure 1:**
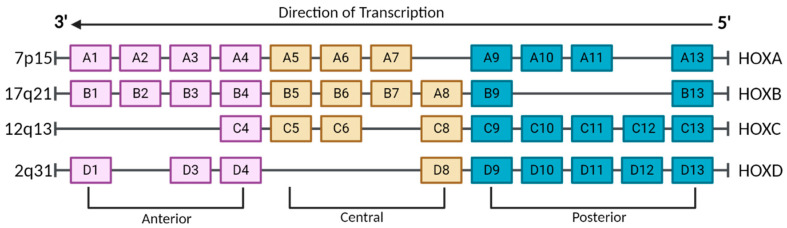
HOX gene family clusters on each corresponding chromosome, with transcriptional direction and anterior and posterior expression noted. Figure created with BioRender (https://biorender.com accessed on 14 October 2024).

The homeobox (HOX) gene family encodes for highly conserved transcription factors that are key regulators in human embryonic development. These genes were first discovered in *Drosophila melanogaster* to function as master regulators of the development of each segment of the fly and, therefore, coined “homeotic selector genes” [1,2,3]. A total of 39 human HOX genes are categorized into 4 clusters: A, B, C, and D, which are located on different chromosomes at 7p15, 17q21, 12q13, and 2q31, respectively (Figure 1). For most of the genome, the arrangement of genes on chromosomes does not correlate to the positional expression within the body. However, the HOX genes are specifically correlated to positional expression, dependent on chromosomal order and location, known as the anterior–posterior axis [4,5]. For example, genes that control the formation of upper-body development are clustered on the 3′ or anterior end of the chromosome, while genes that regulate lower-body formation are on the 5′ posterior end, with genes that are centrally located on the chromosome governing development of the center of the body [6,7,8].

Human HOX genes are small yet powerful transcription factors. They contain two exons and a single intron, with a DNA-binding homeodomain always present within the second exon [9]. While most HOX downstream targets are ill-defined, it has been shown that HOX genes are crucial to normal embryogenesis, limb placement, and organ development [10,11]. Gene expression is mainly restricted to the anterior–posterior axis to ensure normal development of the embryo; however, some HOX genes have been shown to be expressed into adult life, although this is not completely understood. For example, HOX expression has been seen in neurons, lung, and hematopoietic centers past the embryonic stage, yet the function of these genes in adulthood is under-researched [11]. Many genes and pathways vital to embryonic development have been identified in carcinogenesis; therefore, the HOX genes have been implicated as a link between these two processes [12]. Growing evidence suggests that the dysregulation of HOX genes is a key factor in the development and progression of cancer. Dysregulation can occur through epigenetic modification or temporospatial deregulation, leading to either aberrantly increased or decreased HOX expression [13,14]. Depending on the type of cancer and the type of dysregulation, HOX genes can function as oncogenes or tumor suppressors [15,16]. Dysregulated HOX genes have been shown to have numerous functions in cancer, including differentiation, invasion, epithelial-to-mesenchymal transition (EMT), apoptosis, and receptor signaling [10,11,12,16]. The dysregulated HOX gene protein products aid in carcinogenesis through regulation of multiple signaling pathways, including the Notch pathway, Sonic Hedgehog pathway, Paxillin pathway, Wingless-type MMTV Integration Site Family, and Sry-box transcription factor family members [12,17,18]. Through governance of these signaling pathways, HOX genes and their encoded factors can drive cancer initiation, progression, and outcomes. The multifaceted role of HOX genes in carcinogenesis prompted this narrative review, in which we highlight the novel role of the HOX gene cluster and their encoded factors in cancer detection and patient outcomes across the most studied cancer types within this research area while investigating the mechanisms in which this occurs.

## 2. HOX Gene Family’s Role in Myeloid Leukemia

Leukemias are a group of malignant blood or bone marrow disorders with varying pathologies and clinical outcomes. Acute myeloid leukemia (AML) is an aggressive hematological malignancy that accounts for about 80% of all bone marrow cancers in which genetic changes in hematopoietic precursor cells result in the accumulation of myeloid progenitors [19]. This genetically diverse cancer is most common in adults, with the median age of diagnosis between 60 and 70 years old [20]. Intensive chemotherapy and allogeneic stem cell transplants are common treatments; however, the increased age of diagnosis renders many patients ineligible. AML patients have a five-year survival rate of no more than 50%, with a worse prognosis of less than 20% in elderly patients [21]. Almost 50% of AML patients have a normal karyotype, therefore making advanced treatments that are common for other cancers a challenge [22]. Chronic myeloid leukemia (CML) accounts for about 15% of all leukemias in adults and is marked by a reciprocal chromosomal translocation known as the Philadelphia chromosome [23]. This translocation results in the fusion and expression of the tyrosine kinase genes, BCR/ABL1, related to oncogenic transformation and proliferation [24]. Current treatment includes tyrosine kinase inhibitors (TKIs), allowing most chronic-phase patients a near-normal life expectancy [25]. However, some patients become TKI resistant, allowing this disease to progress to blast-phase CML, in which the only treatment is stem cell transplantation [26]. Thus, it is vital to identify novel biomarkers for early detection and prognostic significance to treat patients with these malignancies accordingly.

### 2.1. Role of HOXA10 in Acute Myeloid Leukemia

A study by Guo et al. explored the promising prognostic significance of HOXA10 in AML [21]. The HOXA gene cluster plays a crucial role in adult hematopoiesis; however, aberrant expression promotes oncogenesis [27]. Using bioinformatics, HOXA10 was found to be overexpressed in AML patients compared to a control group consisting of unsorted and CD3+ bone marrow cells. HOXA10 was also significantly enriched in AML patients compared to patients with other myeloid malignancies, therefore indicating HOXA10’s function as a prognostic indicator may be AML specific. HOXA10 shows promise as a prognostic indicator in AML—patients with high HOXA10 expression had more advanced risk stratification and shorter survival times. Kaplan–Meier survival plots demonstrated that both the median overall survival and disease-free survival of AML patients with HOXA10 overexpression were significantly reduced when compared to patients with low expression. Previous research conducted by Thorsteinsdottir et al. has shown that retroviral overexpression of HOXA10 leads to the formation of AML through the alteration of differentiation of murine myeloid progenitor cells [28]. Genome-wide expression profiles of AML patients with high HOXA10 expression compared to low expression were undertaken to elucidate the genetic signature related to poor prognosis in these patients [21]. In silico analyses revealed several pathways significantly correlated with HOXA10 expression that were either suppressed or activated, most notably, the dysregulation of reticular activation system (RAS) and phosphatidylinositol 3-kinase (PI3K)-AKT signaling pathways, as well as activation of ribosomal, oxidative phosphorylation (OXPHOS), and lysosomal pathways. Interestingly, nine other HOX family genes were upregulated in patients with high HOXA10, therefore indicating, with further investigation, a potential HOX family genetic signature of AML. Dysregulation of the RAS signaling pathway is frequently seen in AML patients, with activating mutations and hyperactive mutations promoting cancer development and oncogenesis [29]. Specifically, RAS signaling genes, protein tyrosine phosphatase non-receptor type 11 (PTPN11), FMS-like tyrosine kinase 3 (FLT3), and KIT, were correlated with high HOXA10 expression, thus contributing to the poor prognosis of high-HOXA10-expressing AML patients. Furthermore, P13K-AKT signaling was repressed in patients with HOXA10 overexpression. The P13K-AKT pathway is commonly upregulated in AML cells, regulating glucose metabolism and glycolysis, which are key functions for controlling blast cell proliferation and clonogenicity [30,31]. Previous research has shown that patients with constitutively active PI3K-AKT genes have significantly longer overall survival [32]. Therefore, the low survival rate in AML patients with high-HOXA10 may be attributed to the downregulation of PI3K-AKT signaling. Ribosome-related genes, eukaryotic translation initiation factor 2 alpha kinase 2 (EIF2AK2) and 3 (EIF2AK3), were significantly upregulated in patients with high HOXA10. EIF2AK2 and EIF2AK3 contribute to reduced overall survival of high HOXA10 AML patients via reducing the DNA damage response and promoting dissemination of leukemia cells, respectively. AML cells rely on OXPHOS for energy instead of glycolysis seen in normal hematopoietic cells [33]. Past studies have indicated that increased OXPHOS in AML induces chemotherapeutic resistance [34,35]. Therefore, HOXA10 could not only serve as a prognostic indicator but also as a potential biomarker for chemoresistance in AML patients.

### 2.2. Role of HOXB5 in Acute Myeloid Leukemia

According to Chen et al., bioinformatics analysis of patients with AML and healthy volunteers indicated that HOXB5 was significantly overexpressed in AML patients [22]. Using TCGA and VIZOME databases, HOXB5 was revealed to be associated with leukocytosis and hyperleukocytosis, both indicators of poor prognosis. Increased HOXB5 expression was found to be associated with a worse cytogenetic risk and decreased overall survival. The AML dataset was then analyzed for four genes, DNA methyltransferase 3 alpha (DNMT3A), FLT3, nucleophosmin 1 (NPM1), and runt-related transcription factor 1 (RUNX1), commonly mutated in AML. Mutations in these genes have been associated with worse outcomes in other studies [36,37,38]. Patients with high HOXB5 expression had higher mutation frequencies in DNMT3A, FLT3, and NPM1. Further analysis indicated that HOXB5 was associated with the tumor necrosis factor/nuclear factor kappa-light-chain-enhancer of activated B cells (TNF/NF-kB) pathway and myeloid differentiation. The TNF/NF-kB pathway is essential for maintaining AML proliferation while acting to prevent apoptosis [39]. In vitro knockdown of HOXB5 resulted in the downregulation of TNF-α, p-RELA, and p-IκBα, which are key TNF/NF-κB markers. NF-κB genes, SPI1, interleukin 1 beta (IL-1β), and interleukin 6 (IL-6), were also decreased in HOXB5 knockdown cells. It has been shown that NF-κB activation is associated with leukemia stem cell (LSC) formation, and a positive feedback loop exists between TNF-α secretion and NF-κB activation [40]. Flow cytometry revealed that HOXB5 knockdown cells expressed myeloid differentiation markers CD11b and CD14, indicating that HOXB5 knockdown could promote differentiation. Therefore, these findings indicate that HOXB5 may play a role in LSC maintenance, thus contributing to the poor prognosis in patients with HOXB5 overexpression.

### 2.3. Role of HOXA5 in Chronic Myeloid Leukemia

Strathdee et al. indicated that HOXA5 hypermethylation was associated with poor prognosis in CML [41]. Hypermethylation causes transcriptional repression, therefore downregulating the expression of HOXA5. Aberrant hypermethylation has been previously described to be a key factor in the transformation of normal cells into cancer cells [42]. Chronic-phase CML patients, those at high risk of blast crisis, frequently had HOXA5 hypermethylated, having more than 80% methylation of promoter CpG sites, and had HOXA4, found via the COBRA method and pyrosequencing. As chronic-phase disease is marked by blast crisis, in which differentiation is arrested, the repression of HOXA5 by hypermethylation is proposed to be a key factor due to the HOX genes’ role as regulators of hematological differentiation [27]. Both HOXA5 (91% of patients) and HOXA4 (87%) hypermethylation are associated with high-risk patients and progression into blast crisis. No other HOXA gene was hypermethylated, therefore indicating specificity to these two genes. In vitro re-expression of HOXA5 was shown to induce myeloid cell differentiation, as seen in the expression of CD13, CCAAT/enhancer-binding protein-alpha (C/EBPa), and CCAAT/enhancer-binding protein-epsilon (C/EBPe). This indicates that HOXA5 is a key mediator of differentiation associated with the granulocytic lineage. A different study conducted by Elias et al. showed that aberrant methylation of HOXA4 and HOXA5 has been associated with imatinib resistance in CML patients [43], thus confirming that the hypermethylation of these genes correlates with a poor prognosis outcome. Overall, HOXA5, and potentially in combination with HOXA4, hypermethylation could be utilized as a prognostic biomarker for chronic-phase patients at high risk for blast cell crisis and imatinib resistance, therefore indicating poor patient prognosis.

## 3. Multiple Myeloma

Multiple myeloma, a clonal plasma cell proliferative disorder, is the second most common hematological malignancy. This neoplasm is characterized by an abnormal increase in monoclonal immunoglobulins and originates in post-germinal B lymphoid cells. Multiple myeloma is considered a stage in the spectrum of monoclonal gammopathy that is thought to arise from a premalignant phase of clonal plasma cell growth referred to as monoclonal gammopathy of undetermined significance (MGUS) [44]. MGUS occurs in an asymptomatic phase and is defined as the detection of monoclonal immunoglobulins in the blood or urine without evidence of organ damage [45]. However, there is notable morbidity if left unchecked, as specific end-organ damage often occurs. Frequent alterations and translocations of genes on chromosome 14 lead to disease development, and oncogenes, such as KRAS, NRAS, and BRAF, have been shown to play roles in uncontrolled plasma cell proliferation. Multiple myeloma is relatively uncommon, accounting for less than 2% of diagnosed cancer cases in the United States each year. It most commonly affects the geriatric, male, and African American populations, with a median age of 70 at the time of diagnosis, a 1.4:1 ratio of males to females, and two-fold more incidence in African Americans as compared to Caucasians [46]. Within the past decade, multiple advances have been made in the diagnosis and treatment of multiple myeloma, leading to better staging criteria and improved overall survival. Traditional treatments, such as chemotherapy, radiation, and bone marrow transplants, are common for patients with multiple myeloma. In addition, novel immunotherapies, such as CAR-T-cell therapy, have been recently used to treat multiple myeloma patients [44]. Although several effective therapies exist for the treatment of multiple myeloma, the use of HOX genes as potential biomarkers and therapeutic targets may be of great benefit [47].

### 3.1. Role of HOXB7 in Multiple Myeloma

A study conducted by Storti et al. found that HOXB7 expression is a key mediator of angiogenesis in MM patients [48]. A subset of MM patients expressed HOXB7 in primary tumors, of which all had increased bone marrow angiogenesis marked by upregulation of the pro-angiogenic genes matrix metallopeptidase 2 (MMP2) and platelet-derived growth factor subunit A (PDGFA). Angiogenesis of the bone marrow is associated with MM progression, thus contributing to a worse prognostic outcome for these patients [49,50]. In vitro expression of HOXB7 in JJN3, a cell line that does not constitutively express this gene, resulted in the significant expression of the pro-angiogenic markers vascular endothelial growth factor A (VEGF-A) and fibroblast growth factor 2 (FGF2). HOXB7 overexpression also increased chemokines CCL3 and CCL5. These chemokines promote angiogenesis in response to inflammatory stimuli, aiding in cancer progression [51]. These findings were confirmed in vivo, where HOXB7 overexpression increased tumor growth and stimulated bone marrow vascularization. The results of this study concluded that HOXB7 expression in MM patients correlates with bone marrow angiogenesis, a marker of MM progression. A previous study conducted by Colla et al. explored the link between bone marrow angiogenesis and HOXB7 in multiple myeloma patients [52]. Therefore, HOXB7 can be used as a prognostic indicator for advanced-stage disease while identifying patients who may benefit from treatments with anti-angiogenic properties.

### 3.2. Role of HOXC6 in Multiple Myeloma

HOXC6 overexpression in patients with MM has been indicated as a prognostic marker by Li et al. [53]. HOXC6 mRNA and protein levels were significantly increased in all MM patients’ peripheral blood that was studied. Kaplan–Meier analysis revealed patients with high levels of HOXC6 had significantly decreased overall survival. HOXC6 expression was significantly associated with a decreased hemoglobin level and increased ISS stage, which are both indicators of disease progression [54]. In vitro silencing of HOXC6 was revealed to decrease cell viability and proliferation while promoting apoptosis through caspase 3 and caspase 9 activity. Inflammatory mediators TNF-α, IL-6, and IL-8 were significantly reduced by HOXC6 silencing. TNF-α has been identified as a crucial survival factor in cytokine-dependent (i.e., IL-6) myeloma [55]. Therefore, HOXC6 could not only be used as a prognostic indicator but also as a biomarker to identify patients for anti-TNFα therapies.

## 4. Thyroid Cancer

Thyroid cancer (TC) is the most common endocrine malignancy, accounting for about 3% of worldwide diagnosed cancer cases each year. There are various diagnostic and staging criteria due to the several distinct subtypes that exist due to differences in the degree of differentiation [56]. Well-differentiated TC, including follicular (FTC) and papillary thyroid cancer (PTC), account for about 10–20% and 80% of cases, respectively. Undifferentiated thyroid cancer, including anaplastic thyroid cancer (ATC), accounts for less than 2% of cases and is a much more aggressive and lethal subtype of this endocrine malignancy [57]. FTC and PTC cases have higher increased survival rates due to better responses to existing treatments; however, disease recurrence remains a prominent issue, as these subtypes typically affect younger populations. Well-differentiated TC is staged using the typical staging system of I–IV, whereas undifferentiated TC is diagnosed as Stage IV metastatic disease with further breakdown into Stage IVA, IVB, or IVC. Current diagnostic techniques for identification of TC include fine-needle aspiration, but this technique fails to differentiate between FTC and PTC. Common driver mutations in all TC subtypes include the BRAF point mutation as well as other mutations in the MAPK cascade [58]. Although this is well studied, these mutations alone are not sufficient enough for early diagnosis or disease prognosis. Therefore, the identification of novel biomarkers and therapeutic targets, including the HOX family of genes, in thyroid cancer progression and development may be of significant interest.

### 4.1. Role of HOXD10 in Papillary Thyroid Cancer

Cao et al. investigated HOX gene expression through the use of immunohistochemistry (IHC) in patient PTC tissue [59]. This study consisted of 98 patients in whom HOXD10 expression was found to be decreased in PTC tissue compared to para-cancer tissues using IHC. In silico analysis revealed that decreased expression of HOXD10 was associated with lymph node metastasis and extrathyroidal extension in the PTC patients, both of which are markers of disease progression and poor prognosis [60,61]. There has been growing evidence to suggest that HOXD10 functions as a tumor suppressor. For example, in vitro studies in other cancer types have shown that reduced expression of HOX10 activates extracellular signal-regulated kinases (ERK) signaling and promotes proliferation and aggressive cancer phenotypes [62,63]. On the other hand, one study demonstrated that the reintroduction of HOXD10 resulted in a significant reduction in cell survival, cell migration, and metastasis while increasing apoptosis [64]. While the role of HOXD10 in PTC is currently unknown, evidence supports that HOXD10 has a crucial role as a prognostic indicator while also functioning as a potential tumor suppressor gene in PTC, however further studies are warranted.

### 4.2. Role of HOXD9 in Anaplastic Thyroid Cancer

A study conducted by Zhong et al. reported that HOXD9 is significantly upregulated in anaplastic thyroid cancer and correlated with poor prognosis [65]. Both HOXD9 mRNA and protein were found to be significantly elevated in most ATC tumor tissues when compared to normal tissues via bioinformatics analysis. ATC patients with high expression of HOXD9 had a poorer prognosis compared to patients with low HOXD9 expression. A linear regression analysis was used to determine the correlation between HOXD9 expression and pathological characteristics, where high HOXD9 expression was positively correlated with the TNM stage, tumor size, and distant metastasis. HOXD9 levels correlated with proliferation markers proliferating cell nuclear antigen (PCNA) and antigen Kiel 67 (Ki-67), as well as cell cycle genes cyclin A2 (CCNA2) and cyclin B1 (CCNB1), therefore indicating that HOXD9 promotes ATC tumorigenesis [66,67]. Knockdown of HOXD9 resulted in a significant decrease in cell viability, cell migration, invasion, and the ability to form colonies. On the other hand, HOXD9 overexpression led to significantly increased cell proliferation, cell migration and invasion, and reduction of apoptosis. Further Western blot analysis revealed that the oncogenic activity of HOXD9 is related to epithelial-to-mesenchymal transformation, specifically through the modulation of E-cadherin, N-cadherin, and PI3K/AKT signaling to induce a mesenchymal phenotype that results in malignant dissemination [68]. This study’s comprehensive analysis using bioinformatics, in vitro, in vivo, and human tissue samples revealed the potential of HOXD9 as a diagnostic and prognostic biomarker in ATC.

### 4.3. Role of HOXA9 in Papillary Thyroid Cancer

HOXA9 was found to contribute to PTC tumorigenesis in a study by Jin et al. [69]. Increased expression of HOXA9 enhanced calcification, migration, and invasion in PTC in vitro. Calcifications seen in PTC have been associated with increased tumor size, lymph node metastasis, and a more aggressive phenotype [70,71]. This study suggests that the molecular mechanism of HOXA9-mediated carcinogenicity is due to the positive regulation of oncogene Runt-related transcription factor 2 (RUNX2). It has been shown that RUNX2 expression is increased in thyroid cancer tissues compared to patient-matched controls, and this gene correlates with increased invasiveness [72]. RUNX2 has also been identified as a regulator of EMT, thus the enhanced expression of this gene due to positive regulation from HOXA9 results in a more aggressive PTC phenotype [73]. HOXA9 was found to promote calcification, migration, and invasion in PTC, both in a RUNX2-dependent and independent manner. Therefore, HOXA9 can be used as a novel prognostic indicator to identify patients at risk for thyroid calcification and advanced PTC.

## 5. Breast Cancer

Breast cancer is among the most commonly diagnosed malignancies in females each year and accounts for about 30% of female cancers in the United States. About 66% of breast cancer cases will be diagnosed at a localized stage, however, metastasis remains a prominent issue for many cases, with primary metastatic sites including the bones, lungs, and brain. Metastasis at these sites creates several challenges for effective treatment; therefore, diagnosis before metastatic spread is key to proper treatment efficacy and better patient prognosis. Despite a yearly increase in breast cancer incidence of about 5%, there is a five-year survival rate of about 80%, largely due to routine examinations such as mammography and MRI. Most breast cancers are considered sporadic, over 90%, meaning that only 5–10% of cases have an identifiable driver gene mutation [74]. The most common genetic mutations present in patients with this malignancy are the breast-cancer-associated genes 1 and 2, BRCA1 and BRCA2. BRAC1 and BRCA2 are identified as the most high-risk oncogenes and functionally encode tumor suppressor proteins that drive cell cycle immortality [75]. Another significant gene of interest in this malignancy is the human epidermal growth factor-2 (HER2) protein, which plays a significant role in the propagation and progression of breast cancer [76]. There are several molecular subtypes of breast cancer based on a variety of genetic, environmental, and hormonal factors. These subtypes include luminal A, luminal B, basal-like, and HER-enriched. Luminal A and luminal B are both hormone-receptor-positive, however, they differ in their human epidermal growth factor receptor-2 status, with luminal A being HER-2-negative and luminal B being HER-2-positive. Basal-like is both hormone-receptor- and HER-2-negative and HER-enriched is HER-2-positive but hormone-receptor-negative [74,77]. Hormone status is also a highly significant factor in breast cancer diagnosis, with the estrogen receptor and progesterone receptor both playing a role in the proper treatment protocol for the differing status among patients. Cases that lack all three hormone receptors are collectively referred to as triple-negative breast cancer and have the lowest survival rate among all breast cancer patients. This is due to a significant portion of breast cancer treatment being hormone- and hormone-receptor-based [78,79]. Therefore, there is a significant need for the continuing study of potential biomarkers and therapeutic targets that are not dependent upon hormones and hormone receptors, which opens up an avenue of exploration among genes, such as the HOX family of genes, which have been shown to play a role in breast cancer development.

### Role of HOXC8 in Breast Cancer

The role of HOXC8 in breast cancer stem cells (CSCs) was explored by Shah et al. [80]. Breast CSCs were isolated from normal mammary cells as well as all breast cancer subtypes via flow cytometry. HOXC8 was significantly decreased in all breast CSC types compared to normal mammary stem cells. In silico analysis of breast cancer patient datasets confirmed HOXC8’s significant repression in primary breast cancer tumors when compared to non-cancerous tissue. However, there was no significant difference in HOXC8 expression between metastatic tumors and normal tissue, thus indicating that decreased HOXC8 expression is important for breast cancer transformation and initiation, indicating that HOXC8 could be a potential biomarker for early detection. Bisulfite sequencing revealed that the low expression of HOXC8 in breast CSCs is due to methylation of the promoter region. In silico analysis using TCGA breast cancer patient data confirmed the significant increase in DNA methylation at the HOXC8 promoter region in breast cancer tumors compared to normal tissues. In vitro overexpression of HOXC8 in breast CSCs reduced stemness by inducing differentiation into epithelial cells, as seen by flow cytometry analysis of CD44/CD24 fractions and reduced expression of aldehyde dehydrogenase (ALDH1), where ALDH1 is a marker of stem-cell-like characteristics and associated with poor survival in breast cancer patients [81]. Breast CSCs with HOXC8 overexpression were more sensitive to chemotherapy agents, had reduced self-renewal capacity, and had reduced anchorage independence, all indicating the role of HOXC8 in breast cancer initiation [82,83]. In vitro silencing of HOXC8 in non-tumorigenic mammary epithelial cells resulted in increased stemness, self-renewal, and anchorage independence, thus suggesting that HOXC8 could be a factor in transforming normal breast cells into highly plastic CSCs. Therefore, these results indicate that low HOXC8 expression/HOXC8 methylation has the potential to be a marker for early detection of breast cancer regardless of subtype.

## 6. Ovarian Cancer

Ovarian cancer (OC) is the fifth most deadly cancer in women worldwide as well as the most severe form of gynecological cancer. OC has an incidence of 3.4%, affects approximately 300,000 women annually, and has a 30% survival rate [84]. This poor patient prognosis is heavily reflected by the current absence of accurate diagnostic screening or narrative biomarkers. Currently, the only biomarker used for OC diagnosis is cancer antigen 125 (CA125), which, even so, is extremely limited for usage due to its low specificity [85]. Only about 50% of patients in the early stages of OC express CA125, as well as more than 50% of patients with high CA125 do not have OC, making CA125 an unreliable biomarker [86]. Other instances of elevated CA125 are seen in a plethora of other malignancies, such as breast cancer, uterine cancer, colon cancers, and other inflammatory diseases [87,88]. The need for OC-specific markers is extremely pertinent—markers for diagnostic, prognostic, and/or therapeutic value will significantly contribute to combating OC. The embryonic development of the female reproductive system is orchestrated by a series of genes, including many *HOX* family genes [89]. Four *HOX* genes, *HOXA9*, *HOXA10*, *HOXA11*, and *HOXA12*, are reported to have extensive roles in the embryonic development of the female reproductive system, and subsequently play a role in the extensive structural and functional uterine (*HOXA11*), cervical (*HOXA12*), and vaginal (*HOXA13*) changes throughout the female lifetime [90,91]. It is reported that *HOX* genes, when overexpressed, can contribute to the development of ovarian neoplasia due to their significant role as differentiation drivers [92,93]. Moreover, HOXA7 was found to be overexpressed in OC tissues that contain the Mullerian-like phenotype, and HOXA7 can induce this phenotype by upregulating *HOXA9*, *HOXA10*, and *HOXA11*, suggesting a specific prognostic or diagnostic role for individual HOX genes in OC [94]. Therefore, studying HOX genes in OC can lead to successful biomarker discovery, which, in turn, will combat the main hurdle with OC and ameliorate the diagnostic burden.

### 6.1. Role of HOXB13 in Ovarian Cancer

The role of HOXB13 in ovarian cancer was explored in a study conducted by Yuan et al. [95]. In a search to find genes associated with EMT and aggressive phenotypes in ovarian cancer, siRNA inhibition of HOXB13 resulted in the restoration of cell–cell adhesion through the re-expression of E-cadherin. HOXB13 silencing resulted in mesenchymal-to-epithelial transition (MET), in which E-cadherin was upregulated while N-cadherin and vimentin were downregulated. The induction of MET resulted in decreased cell invasion, implicating the key role HOXB13 plays in EMT in ovarian cancer and, therefore, is a promising biomarker for early detection. HOXB13 does not function alone. HOXB13 can form a heterodimer with homeobox protein aristaless-like 4 (ALX4) to induce the expression of transcription factor Snail family transcriptional repressor 2 (SLUG). SLUG has been identified as a key factor in OSC progression, metastasis, and angiogenesis, all characteristic of worse patient prognosis [96]. Additionally, HOXB13 has been shown to enhance breast cancer proliferation and decrease apoptosis while also contributing to tamoxifen resistance, widely used for recurrent OSC [97]. Overall, the use of HOXB13 as a prognostic indicator for advanced disease and tamoxifen resistance is promising due to this gene’s role in cancer progression.

### 6.2. Role of HOXD4 in Ovarian Cancer

Yu and Guo revealed the prognostic significance of HOXD4 in ovarian cancer [98]. HOXD4 protein expression in OSC tissue samples was compared to patient-matched non-cancerous adjacent tissue by IHC. OSC tissues had enriched HOXD4 levels compared to normal tissue, which was confirmed by increased mRNA levels in OSC patients as well. High HOXD4 levels correlated with lymph node metastasis and an increased FIGO stage. Kaplan–Meier survival analysis showed that HOXD4 expression correlated significantly with decreased survival and was confirmed as an independent risk factor of OSC progression by multivariate analysis. In vitro silencing of HOXD4 reduced cell proliferation and survival, thus suggesting the function of HOXD4 as an oncogene in OSC. The mechanism by which HOXD4 drives oncogenesis has yet to be explored and requires further examination. However, the use of HOXD4 as a prognostic biomarker allows healthcare professionals to take a more aggressive approach to treatment in patients with high expression of this gene.

## 7. Gastric Cancer

Gastric cancer (GC) is the fourth leading cause of cancer-related deaths worldwide and the fifth most common cancer worldwide. There are several types of GCs, the most common being gastric adenocarcinoma, with less common types such as gastrointestinal stromal tumors (GIST), carcinoid tumors, and gastroesophageal junction adenocarcinoma (GEJ). Risk factors include *Helicobacter pylori* infection, consumption of highly processed or salty foods, obesity, and smoking. Current treatments for this malignancy include surgical resection, chemotherapy, radiation, and in some cases, immunotherapy. Despite advances in detection and therapeutic intervention, prognosis remains poor, with early-stage disease only accounting for 10% of all GC cases [99]. With a five-year survival rate of 30% for regional GC and less than 5% for metastatic disease, there is a great need for biomarker discovery [100]. Currently, there are three biomarkers used for GC diagnosis: human epidermal growth factor receptor 2 (HER2), microsatellite instability-high (MSI-H), and programmed death-ligand 1 (PD-L1) [101]. However, the specificity of these biomarkers is low due to intra-tumoral heterogeneity and high discordance rates, with about 5–25% of GCs having overexpression of any of the three biomarkers [102]. With the overall poor prognosis for GC patients and lack of effective biomarkers, the role of HOX genes in GC progression and development is of significant interest.

### 7.1. Role of HOXA10 in Gastric Cancer

HOXA10 was associated with the gastric cancer phenotype in a study conducted by Sentani et al. [103]. Microarray and IHC analysis of patient samples revealed an upregulation of HOXA10 in GC tissue compared to normal gastric mucosa. In silico analysis indicated that HOXA10 expression was correlated with a decreased depth of invasion and increased patient survival. Differentiated GC, specifically Mucin 2 (MUC2)-positive tissues, significantly correlated with HOXA10 expression. Goblet cells of the intestinal tract express MUC2, thus this gene is used to identify the intestinal phenotype of gastric cancer [104]. HOXA10 expression also occurred more frequently in GC tissues that expressed caudal type homeobox 2 (CDX2), in which this gene plays an important role in the regulation of intestinal epithelial cells and has been recognized as a possible GC tumor suppressor gene [105]. In vitro silencing of HOXA10 increased cancer cell viability and invasion, therefore suggesting that HOXA10 functions as a tumor suppressor gene in GC. However, further studies to elucidate the exact mechanism by which HOXA10 mediates the carcinogenic effects in GC is necessary, possibly acting jointly with CDX2. Therefore, HOXA10 has potential as a novel prognostic biomarker for increased survival in patients with intestinal mucin phenotype GC through possible action as a tumor suppressor gene.

### 7.2. Role of HOXB9 in Gastric Cancer

Kato et al. examined the role of HOXB9 in GC, finding that increased expression was associated with a worse patient prognosis [106]. HOXB9 overexpression was identified in about 50% of all GC patient tissue samples studied. In silico analysis of patients with high HOXB9 expression displayed an increased tumor depth, lymph node involvement, and vascular invasion, therefore indicating a worse patient outcome. In vitro overexpression of HOXB9 resulted in increased vascular endothelial growth factor D (VEGF-D) expression, indicating a positive relationship between the two. In GC, VEGF-D has been correlated with distant metastasis and reduced relapse-free survival due to its role in lymphatic and venous invasion [107]. In conclusion, patients with high HOXB9 expression are prone to a more aggressive GC phenotype, marked by increased VEGF-D expression and, therefore, a negative clinical outcome. With that being said, high-HOXB9-expressing patients may benefit from anti-VEGF treatment due to the correlation between these two genes.

### 7.3. Role of HOXA5 in Gastric Cancer

A study conducted by Wu et al. demonstrated that HOXA5 acts as a tumor suppressor in gastric cancer [108]. Thirty GC patient tissues compared to adjacent non-cancerous tissues showed decreased expression of HOXA5 mRNA and protein. IHC confirmed decreased HOXA5 staining, and these results were consistent with in vitro cell lines. HOXA5 downregulation was significantly associated with an increased tumor size and advanced TNM stage. Patients with high HOXA5 expression had significantly better overall survival (OS) and disease-free survival (DFS) via Kaplan–Meier analysis. In vitro overexpression of HOXA5 in gastric cells resulted in decreased cell viability and induced apoptosis, whereas knockdown had the inverse effect. HOXA5 was shown to be regulated by miR-196a through an inverse relationship. miR-196a is transcribed from the HOX loci, and identified as an oncogene in several cancer types, while specifically inducing invasion and metastasis in GC [109]. Taken together, HOXA5 has significant prognostic power in GC.

### 7.4. Role of HOXC9 in Gastric Cancer

An investigation of GC by Zhao et al. revealed that HOXC9 correlates with poor patient prognosis [110]. HOXC9 mRNA and protein were significantly increased in GC tissues vs normal gastric tissue. High HOXC9 expression was correlated with increased tumor size, lymphatic invasion, depth of invasion, lymph node metastasis, and increased TNM stage. Kaplan–Meier curves demonstrated that high HOXC9 expression was associated with decreased OS and DFS. Cox regression revealed HOXC9 as an independent poor prognostic indicator in GC. HOXC9 in vitro silencing resulted in decreased migration and invasion, however, the mechanism by which this occurs remains unexplored. A different study elucidated that HOXC9 functions as an oncogene in GC by modulating the immune response, specifically inhibiting interferon-gamma (IFN-γ)-dependent apoptosis [111]. Therefore, these data conclude that HOXC9 is a powerful prognostic biomarker for patients with increased levels of this gene.

## 8. Renal Cancer

Renal cancer, also known as kidney cancer, is among the top ten most common cancers in males, with a recent rise in incidence. There are several types of kidney malignancies, with renal cell carcinoma (RCC) being the most prevalent, with more than 300,000 new cases every year [112]. RCC has several subtypes that are diagnosed based on pathology, which include kidney renal clear cell carcinoma (KIRC), chromophobe renal cell carcinoma (KICH), papillary renal cell carcinoma (KIRP), and renal collecting duct carcinoma. KIRC, also named clear cell renal cell carcinoma, is the main subtype and comprises more than 80% of all RCC cases [113]. Treatment options include surgical resection, targeted therapies, such as tyrosine kinase inhibitors, and immunotherapies; however, there are limitations due to treatment resistance and challenges of surgical resection in advanced-stage disease. The majority of patients are diagnosed at an advanced stage due to the asymptomatic nature of RCC and the lack of definitive biomarkers [114]. Furthermore, 30% of patients experience tumor recurrence after curative treatment for local RCC [114]. Current biomarkers are associated with rare hereditary diseases, such as VHL and MET gene mutations that play a crucial role in guiding therapy and predicting responses, however, they only occur in about 4% of RCC cases [115]. Therefore, it is essential to explore novel effective biomarkers that can guide therapeutic interventions and improve patient survival.

### 8.1. Role of HOXD1 in Renal Cancer

Cui et al. demonstrated the promising usage of HOXD1 as a prognostic indicator in KIRC [116]. Bioinformatics analysis revealed that HOXD1 was significantly downregulated in KIRC samples compared to normal renal tissue. Low expression of HOXD1 was associated with decreased OS, DFS, and increased TNM stage, therefore indicating that reduced HOXD1 correlates with a worse patient prognosis. In vitro overexpression of HOXD1 resulted in decreased proliferation and cell cycle arrest in KIRC cells by inhibiting transforming growth factor beta (TGF-β) signaling. TGF-β has been implicated in EMT in renal cancer, thus suggesting that HOXD1 functions as a tumor suppressor via inhibition of TGF-β [117]. A different study also found that HOXD1 was significantly decreased in KIRC as well as other RCC types—KICH and KIRP [118]. KIRC and KICH patients with high HOXD1 expression had significantly longer OS as well as improved progression-free survival. Further bioinformatics analysis using TIMER revealed that HOXD1 positively correlated with immune infiltration, thus indicating that HOXD1 could be an indicator for the use of immunotherapeutic agents in patients with RCC.

### 8.2. Role of HOXC11 in Kidney Renal Clear Cell Carcinoma

Bioinformatics analysis conducted by Cui et al. revealed that HOXC11 was significantly upregulated in KIRC patient samples [119]. High HOXC11 expression was correlated with worse OS, and in vitro knockout of HOXC11 resulted in decreased proliferation and apoptosis induction, therefore suggesting HOXC11 functions as an oncogene. Further in silico analysis demonstrated that HOXC11 mediates fatty acid metabolism and mechanistic target of rapamycin kinase (mTOR) pathways, each of which plays a role in carcinogenesis [120,121]. HOXC11 inhibited peroxisome-proliferator-activated receptor gamma (PPARγ), a gene shown to induce apoptosis and cell cycle arrest, therefore suggesting that the oncogenic effects of HOXC11 arise from inactivation of PPAR signaling [122]. A second study by Liu et al. confirmed the prognostic value of HOXC11 [123]. HOXC11 mRNA and protein expression were both significantly increased in RCC compared to normal kidney tissue. In vitro overexpression of HOXC11 in the HK-2 human epithelial cell line fostered proliferation, whereas downregulation of naturally increased HOXC11 levels in human RCC cells had an inhibitory effect. IHC staining of RCC tissues indicated a strong correlation between HOXC11 and Ki67 expression, indicating that HOXC11 promotes tumor proliferation and, therefore, a worse prognosis [124]. Elevated levels of HOXC11 were also attributed to patients with an advanced TNM stage, shorter OS, and shorter progression-free survival than patients with low levels of this gene. Overall, these data suggest that HOXC11 is an important mediator of carcinogenesis in RCC and a novel determinant of patient prognosis.

### 8.3. Role of HOXA13 in Kidney Renal Clear Cell Carcinoma

Cui et al. identified HOXA13 as an oncogene in KIRC [125]. Bioinformatics analysis resulted in HOXA13 significantly upregulated in KIRC vs normal tissues. High HOXA13 expression was correlated with decreased OS and DFS. HOXA13 knockdown in vitro resulted in significant proliferation inhibition through arresting cells in the G0/G1 phase of the cell cycle. The cell cycle arrest seen in HOXA13 knockdown cells was attributed to the increase of tumor suppressor gene P53 and decrease of cell cycle promoter cyclin D1 (CCND1). Overexpression of HOXA13 had the opposite effect, therefore suggesting that HOXA13 acts as an oncodriver through promoting proliferation. A different study confirmed that HOXA13 expression was increased in KIRC tissues via bioinformatics analysis [126]. HOXA13 was found to interact with miRNAs: miR-4668-5p, miR-4768-5p, and miR-25-3p. MiR-4668-5p has been associated with sunitinib resistance in metastatic RCC patients, while miR-25-3p promoted renal cell proliferation through inhibiting autophagy [126,127]. Therefore, HOXA13 shows promise as a prognostic indicator in RCC due to its action as an oncogene.

## 9. Colon Cancer

Colon cancer, also known as colorectal cancer (CRC), is the third most diagnosed cancer globally, with the occurrence in the young population on the rise [128]. Early detection is highly successful, as this malignancy is characterized by the sporadic accumulation of mutations over a period of 10–15 years and pre-cancerous adenomas can be removed, leading to a decrease in CRC incidence and mortality [129]. The standard screening strategy is regular colonoscopies starting at the age of 45, however patient compliance is low due to the invasiveness and risks associated with this procedure [130]. Non-invasive screening using a fecal occult blood test is cost-effective but comes with poor selectivity and sensitivity. Treatment options typically include surgical resection, chemotherapy, radiation therapy, and targeted therapies, targeting EGFR or VEGF pathways. However, there are limitations with these therapeutic interventions that include challenges in resection and therapeutic resistance due to tumor heterogeneity and high mutational burden [131]. Many of the published findings on molecular biomarkers in CRC are considered controversial, with KRAS being the most reliable marker for metastatic patients receiving EGFR therapy [132]. Thus, novel biomarker discovery is crucial for early detection and prognostic stratification to improve patient survival.

### 9.1. Role of HOXD10 in Colon Cancer

Yaun et al. explored the role of HOX10 in colon cancer [133]. Bioinformatics analysis revealed that HOXD10 was reduced in colorectal cancer due to hypermethylation of the HOXD10 promoter. Increased HOXD10 methylation was associated with a non-significant decrease in overall survival based on TCGA datasets. In vitro colon cancer cell lines exhibited the same pattern of HOXD10 hypermethylation subsequent to mRNA repression. Demethylation of HOXD10 in vitro resulted in increased HOXD10 levels, which reduced cell proliferation and induced apoptosis. In vitro overexpression of HOXD10 resulted in the same impact on proliferation and apoptosis, therefore suggesting that HOXD10 functions as a tumor suppressor and, thus, hypermethylation of this gene allows for CRC progression. HOXD10 overexpression resulted in the inactivation of the AKT and ERK/MAPK pathways, thus abrogating carcinogenicity. AKT and the ERK/MAPK pathways are key mediators of CRC, regulating metabolism, EMT, cell cycle progression, and metastasis [134,135,136]. Similarly, HOXD10 expression is inversely related to RAS homolog family member C (RHOC) expression, a gene implicated in metastasis and CRC recurrence [137]. As a result, hypermethylation of HOXD10 or decreased HOXD10 can be a vital diagnostic biomarker for early detection of CRC.

### 9.2. Role of HOXA5 in Colon Cancer

Bioinformatics analysis of colon cancer datasets revealed that HOXA5 was significantly downregulated in both colorectal adenoma and carcinoma when compared to normal colon tissue [138]. These samples exhibited a strong inverse relationship between HOXA5 and leucine-rich-repeat-containing G protein-coupled receptor 5 (LGR5), a Wnt target gene. Wnt signaling is often hyperactive in colon cancer, leading to metabolic reprogramming that is key in cancer stemness and progression [139,140]. This study also demonstrated that HOXA5 was strongly inversely correlated with MYC, where in vitro silencing of MYC increased HOXA5 expression. Increased expression of MYC is a driver of carcinogenesis and is often correlated with hyperactive Wnt signaling [141,142]. Further exploration revealed that patients with high levels of HOXA5 had significantly better relapse-free survival. For that reason, HOXA5 functions as a tumor suppressor gene in CRC and is repressed by MYC and Wnt signaling. HOXA5 expression in colon cancer cell lines induced terminal differentiation and epithelial morphology instead of a stem cell phenotype. Specifically, cancer stem cell markers LGR5, CD44v6, and AC133 drastically decreased with HOXA5 re-expression in vitro, indicating that loss of HOXA5 is imperative to stem cell transformation. Stemness has been associated with worse patient outcomes, immunosuppressive TME, and treatment resistance in CRC patients, thus indicating that decreased HOXA5 expression and its relationship to stemness could identify patients at risk [143]. Overall, HOXA5 has been shown to block stemness in colorectal cells by inhibiting Wnt signaling activity and, as a result, can be useful as a powerful prognostic biomarker for colorectal cancer.

### 9.3. Role of HOXD8 in Colon Cancer

A study published by Mansour et al. identified HOXD8 as a tumor suppressor gene in CRC [144]. Analysis of 18 CRC patients revealed that HOXD8 mRNA levels were significantly decreased in CRC tissue compared to normal tissues. In silico analysis of several datasets showed the same reduction of HOXD8 in CRC. In vitro expression of HOXD8 inhibited proliferation while inducing apoptosis. HOXD8 expression also reduced anchorage independence and cell invasion, two processes fundamental in tumorigenesis. This reduction was due to HOXD8 suppression of paladin and enhancement of E-cadherin. Paladin overexpression has been correlated with decreased survival in CRC due to its function in actin cytoskeleton organization, while decreased expression of E-cadherin, a marker of epithelial integrity, is correlated with shorter OS, metastasis, and decreased DFS in CRC patients [145]. This implies that the loss of HOXD8 seen in CRC is crucial for EMT and cancer progression. Moreover, HOXD8 was inversely related to serine/threonine kinase 38 (STK38) and MYC, two genes related to cancer progression. As a result, HOXD8 is a novel determinant of patient outcomes due to this gene’s role as a tumor suppressor in CRC.

## 10. Melanoma

Melanoma is one of the most aggressive skin cancers, and in the last decade, its incidence has been rapidly increasing. Nearly 92% of all skin cancer diagnoses are melanoma, and it represents 60% of all skin-cancer-related deaths in the United States [146]. Exposure to ultraviolet (UV) light is the major risk factor for melanoma, with sunlight exposure being the main source of UV rays. Other risk factors include light skin color, family or personal history of melanoma, weakened immune system, old age, and being male, according to the American Cancer Society. Melanoma that is detected early, usually presenting as an irregularly shaped mole, will be removed with a wide local excision. However, accumulating mutations caused by these risk factors in melanin-producing cells or melanocytes cause the rapid expansion and proliferation of a malignant tumor that either remains localized or disseminates and metastasizes throughout the body [147]. All other stages of melanoma require immune checkpoint inhibitors (ICIs) or targeted drug therapies, which have significantly improved overall survival rates. Before the use of these therapies, malignant melanoma had a five-year survival rate of less than 5% [148]. ICIs most commonly used to treat malignant melanoma include anti-CTLA-4 or anti-PD-1/anti-PD-L1 treatment [149]. Although modern immunotherapies like ICIs have significantly improved the overall survival of melanoma patients, the effectiveness is limited to approximately 50% of patients [150]. Therefore, there is a crucial need for biomarker discovery in this cancer type to identify risk progression and treatment options for these patients.

### 10.1. Role of HOXC10 in Melanoma

Miao et al. determined that HOXC10 is a key factor in melanoma development [151]. HOXC10 was upregulated in melanoma patient tissue compared to non-cancerous adjacent tissues from a cohort of 60 patients. In vitro melanoma cell lines also exhibited upregulation of HOXC10 mRNA and protein. Silencing of HOXC10 reduced cell viability, proliferation, and clone formation while inducing apoptosis in vitro. HOXC10 was implicated in EMT, as in vitro knockdown of this gene reduced migration, invasion, and EMT markers N-cadherin and Snail. In melanoma, N-cadherin has been shown to promote proliferation and migration, while Snail promotes tumor growth through the promotion of an immunosuppressive TME [152,153]. Endogenous HOXC10 increased SLUG activity, a target of the yes-associated protein/transcriptional coactivator with PDZ-binding motif (YAP/TAZ) pathway. The YAP/TAZ pathway has been indicated in EMT and the invasive phenotype of melanoma [154]. The use of HOXC10 knockdown cells in vivo resulted in a decreased tumor size and decreased lung metastasis. These data conclude that HOXC10 promotes the progression of melanoma through its role in EMT and, therefore, can function as a potential early diagnostic biomarker.

### 10.2. Role of HOXA1 in Melanoma

A study conducted by Wardell-Ozgo et al. implied that HOXA1 is a key driver of melanoma tumorigenesis [155]. In silico analysis of murine metastatic melanoma tumors pinpointed HOXA1 as the most significant pro-invasion gene. Non-metastatic cells ectopically expressing HOXA1 showed increased invasion, formed lung metastasis, and showed stellate protrusions, which indicated an invasive cell morphology. HOXA1 demonstrated pro-invasion capabilities in human melanoma cell lines as well, where immortalized melanocytes that overexpressed HOXA1 had enhanced invasion and invasive morphological changes. These cells also exhibited anchorage-independent growth in vitro and an increased tumor size when implanted in vivo. Transcriptome analysis of HOXA1 endogenous melanoma cells revealed upregulation of TGF-β signaling and downregulation of melanocyte-inducing transcription factor (MITF). The TGF-β pathway has been implicated in many parts of tumorigenesis, including EMT, cell growth, differentiation, and apoptosis [156]. Eighty-four TGF-β signaling genes were associated with HOXA1 expression, indicating that HOXA1 drives melanoma progression through interaction with the TGF-β signaling axis. MITF has been identified as a regulator of normal melanocyte development and differentiation and has demonstrated the ability to inhibit invasion and EMT in melanoma [157]. Therefore, the downregulation of MITF in melanoma cells with enhanced HOXA1 expression may lead to melanocyte de-differentiation and thus an invasive phenotype due to the upregulation of TGF-β activation. Lastly, bioinformatics analysis uncovered that patients with high HOXA1 had a highly invasive melanoma signature and a reduced time to distant metastasis formation. Therefore, HOXA1 is a prognostic biomarker for highly invasive melanoma and metastasis.

### 10.3. Role of HOXC13 in Melanoma

HOXC13 expression has been identified as an important factor in melanoma progression in a study by Cantile et al. [158]. Forty-eight primary melanoma and corresponding metastatic tissue samples were evaluated for HOXC13 expression by IHC and qRT-PCR. Metastatic tissues had significantly increased HOXC13 expression compared to primary melanoma. In vitro HOXC13 expression confirmed this pattern, in which primary melanoma cell lines had moderate HOXC13 expression, while metastatic cell lines had significantly higher expression. The exact mechanism in which HOXB13 plays a role in the metastatic switch in melanoma has yet to be determined and is a vital area of study that needs further exploration. However, HOXB13 expression has been linked with metastatic progression in prostate cancer through lipid accumulation [159,160]. The studies show the prognostic significance of HOXC13 as a biomarker for metastatic melanoma and, therefore, decreased overall survival.

## 11. Lung Cancer

Lung cancer (LC) is the second most common malignancy worldwide, yet it accounts for the most cancer-related deaths according to the World Health Organization. Risk factors for this type of cancer include tobacco use, secondhand smoke, asbestos, alcohol use, ionizing radiation, pulmonary fibrosis, and radiation therapy to treat other malignancies [161]. LC is divided into two histological types: small-cell lung cancer (SCLC) and non-small-cell lung cancer (NSCLC). NSCLC makes up 80–90% of all lung cancer cases, with the majority of newly diagnosed cases being advanced stage with an overall survival rate of 10–15% [162]. NSCLC is further divided into three subtypes: lung squamous cell carcinoma (LUSC), lung adenocarcinoma (LUAD), and large-cell lung cancer (LCLC). While LUSC and LUAD are the most common subtypes of NSCLC, their biological signatures vary greatly, and biomarkers are few and far between [163]. SCLC accounts for approximately 15% of all LC cases but is highly aggressive and has a high propensity for relapse, with a two-year survival rate of less than 5% due to chemoresistance [164]. Typical treatment for early-stage patients includes lobectomy, chemotherapy, radiation therapy, or a combination of these treatment modalities. However, 30–50% of patients will relapse even after complete resection during early-stage disease [165]. Despite advances in early LC identification and the use of standard treatments, the majority of patients are diagnosed at an advanced stage in which the prognosis is poor, with a five-year survival rate of approximately 10% [166]. Therefore, biomarker discovery is essential, as early detection will prolong patient survival, and prognostic indicators are crucial for the identification of specific therapeutic interventions.

### 11.1. Role of HOXA1 in Lung Cancer

Zhang et al. elucidated the role of HOXA1 as an oncogene in NSCLC tissues via RT-qPCR and bioinformatics-based analyses [167]. The cohort of 53 NSCLC samples was collected during surgical resection, with 22 patients having LUSC and 31 patients with LUAD. NSCLC tissue had increased HOXA1 mRNA when compared to normal lung tissue. Expression of HOXA1 was associated with advanced-stage disease and lymph node metastasis. Three databases, TCGA, Oncomine, and GEPIA, were utilized to explore HOXA1 expression patterns across 11 NSCLC datasets. Ten of the eleven datasets matched the RT-qPCR data, in which NSCLC tissue had high HOXA1 levels compared to normal lung tissue. Further bioinformatics analysis revealed that HOXA1 is associated with the P53 signaling pathway, specifically cyclin-dependent kinase inhibitor 2A (CDKN2A) and checkpoint kinase 1 (CHEK1). As these genes are related to cell cycle control and mainly function to prevent carcinogenic advancement, it is suggested that HOXA1 may contribute to dysregulation of the p53 pathway, therefore resulting in tumorigenesis. However, the specific mechanism by which this takes place has yet to be studied [168]. This study hypothesized that the overexpression of HOXA1 is essential for NSCLC oncogenesis and a promising diagnostic biomarker.

A different study conducted by Xiao et al. showed the overexpression of HOXA1 in SCLC patients [169]. Immunohistochemistry was performed on tissues collected from 63 SCLC patients, where 46% of the patients were HOXA1-positive. Patients with positive HOXA1 staining had a significantly increased overall survival and enhanced chemotherapy response. Chemotherapeutic-resistant SCLC cells exhibited a significant downregulation of HOXA1 in vitro when compared to chemoresponsive SCLC cells. Overexpression of HOXA1 in chemotherapeutic-resistant SCLC cells significantly reduced cell proliferation and survival, as well as reduced the IC_50_ of chemotherapy needed for treatment. On the other hand, HOXA1 silencing in SCLC resulted in chemoresistance and increased cell proliferation and survival. Expression of HOXA1 was inversely correlated with miR-100 and, after further analysis, it was found that HOXA1 is a direct target of miR-100. MiR-100 has been implicated as a tumor suppressor gene by abrogating invasion and migration capacities in NSCLC; however, the role of this miRNA in SCLC appears to have a different role and needs further investigation [170]. Overall, this study implies that HOXA1 is a positive predictor of increased survival and chemotherapeutic response in SCLC.

### 11.2. Role of HOXA13 in Non-Small-Cell Lung Cancer

Evaluation of HOXA13 in NSCLC by Wang et al. revealed that expression of this gene is elevated in malignant tissue compared to normal lung tissue [171]. This upregulation of HOXA13 was demonstrated by qRT-PCR, IHC, and bioinformatic analysis of NSCLC patients. Patients with high HOXA13 had a worse OS and DFS and were determined to have an independent unfavorable prognostic factor via Cox regression. In vitro overexpression of HOXA13 resulted in increased proliferation, while knockdown of this gene inhibited NSCLC growth. qRT-PCR screening and IHC revealed that HOXA13 exerts its oncogenic properties by reducing P53 signaling while stimulating the Wnt/β-catenin pathway. Specifically, P53, a tumor suppressor gene, and its targets, P21, phorbol-12-myristate-13-acetate-induced protein 1 (PMAIP1), and BCL-2-associated X protein (BAX), were inhibited by HOXA13 expression, thus allowing oncogenic proliferation to occur [172]. HOXA13 expression increased β-catenin, and its downstream MYC, CCND1, and matrix metalloproteinase 7 (MMP7) have been shown to promote EMT, metastasis, and poor patient prognosis in NSCLC [173,174]. Further to this point, mice implanted with stably expressing HOXA13 cells had a significantly increased tumor volume as well as increased lung and liver metastasis. Thus, HOXA13 shows promise as a predictor of unfavorable patient outcomes due to this gene’s role in tumorigenesis.

### 11.3. Role of HOXB3 in Lung Adenocarcinoma

A study by Yan et al. revealed that high expression of HOXB3 plays a key role in oncogenesis in LUAD [175]. In silico analysis indicated that HOXB3 was significantly upregulated in LUAD tumors when compared to normal tissue and was a risk factor for decreased survival regardless of tumor stage. HOXB3 was also correlated with shorter relapse-free survival in LUAD patients. Interestingly, the association of high HOXB3 expression and decreased OS and DFS was only significant in females. Using bioinformatics, HOXB3 expression was positively correlated with ten different immune checkpoint molecules, therefore indicating that immune checkpoint blockade (ICB) therapy may be beneficial for these patients in addition to standard treatment. A negative correlation was exhibited between HOXB3 and the quantity of tumor-infiltrating lymphocytes (TILs) and tumor immune stimulators. High levels of TILs have been associated with increased patient prognosis, therefore suggesting that HOXB3 mediates tumor immunity in LUAD [176]. Additionally, HOXB3 is implicated in oncogenic maintenance through modulation of apoptotic pathways. Specifically, HOXB3-silencing-induced apoptosis decreased BCL-2 and increased BAX expression. In conclusion, HOXB3 functions as an oncodriver in LUAD and is a promising novel marker for worse patient prognosis.

## 12. Glioblastoma

Glioblastoma (GBM) is the most common primary brain cancer and is very aggressive and fast growing. GBM has a median survival of approximately 15 months and a 5-year survival of less than 7% [177]. Diagnosis consists of tumor biopsy for histological and molecular evaluation, specifically looking for irregular-shaped tumor cells and the expression of mutations like TP53, PTEN, IDH1, TERT, and EGFR overexpression [178,179]. The current standard treatment for GBM includes surgical resection of the tumor and radiotherapy with adjuvant temozolomide (TMZ), yet GBM continues to have low survival rates due to its refractory nature [180,181]. Major obstacles in treating GBM are related to the invasive nature of this cancer type, difficulty in overcoming the challenges posed by the blood–brain barrier, intra-tumoral heterogeneity, and the immunosuppressive microenvironment [182]. Tumor borders are diffuse, making total surgical resection difficult, and any cells that have invaded and recolonized in healthy tissue may cause treatment-resistant growth, while the high levels of immunosuppressive cells limit the efficacy of immunotherapy [183]. There are several emerging therapies, including immune checkpoint blockade, CAR-T-cell therapy, oncolytic virotherapy, vaccine therapy, and focused ultrasound therapy, however, similar challenges in treatment efficacy still occur [180,184]. There is great potential in the research evaluating aberrant expression of the HOX family in glioblastoma, as HOX genes are important in the development of the brain during fetal development.

### 12.1. Role of HOXC6 in Glioblastoma

A comprehensive study by Eryi et al. investigated the role of HOXC6 in glioblastoma [185]. Using GBM patient gene expression profiles from ONCOMINE and TCGA, HOXC6 expression was revealed to be significantly upregulated in GBM patients compared to healthy controls. Increased expression of HOXC6 was associated with low survival rates and a high risk ratio. IHC staining of GBM patient tissue for HOXC6 resulted in most GBM patients showing HOXC6 expression, whereas no expression was seen in normal control tissues, thus confirming the results seen in the bioinformatic analysis. Glioma cell lines U251 and U97 were used for in vitro experimentation, as HOXC6 expression was the highest in these cell lines. Knockdown of HOXC6 using RNA interference resulted in a significant reduction in proliferation and colony formation. HOXC6 knockdowns also had impaired migration and invasion evaluated via transwell cell migration/chamber invasion assays and the scratch wound-healing assay. In vivo implantation showed that HOXC6 knockdown cell lines had decreased growth and tumor volume. These results led to the investigation of HOXC6’s role in EMT. E-cadherin, an epithelial marker, was significantly upregulated, while N-cadherin and vimentin, markers of mesenchymal cells, were significantly downregulated in the knockdown cell lines, thus indicating that HOXC6 expression regulates EMT in GBM. HOXC6 modulates EMT through the TGF-B/Smad pathway in this cancer type, as HOXC6 knockdowns have decreased TGF-B1, TGFB2, Smad4, and p-Smad2 expression. Therefore, this implicates that the increased expression of HOXC6 in GBM patients regulates the proliferative, migratory, and invasive capacity through the TGFb/Smad pathway. Previous studies have shown that HOXC6 overexpression in GBM promotes proliferation and migratory capacity; however, the specific mechanisms differ, as one study indicated MAPK signaling as the mechanism [186] and another study implicated the WNT pathway [187]. As HOX genes are transcription factors, multiple downstream pathway involvement is not unusual. Overall, HOXC6 shows promise as a prognostic indicator for poor survival in patients with high HOXC6 expression.

### 12.2. Role of HOXD10 in Glioblastoma

A study by Li et al. identified HOXD10 as an indicator of poor prognosis in GBM [188]. Bioinformatic analysis of GBM patient datasets showed that HOXD10 mRNA expression was significantly increased in GBM patients compared to normal tissue. Patients with high HOXD10 expression had a decreased rate of survival via Kaplan–Meir curve analysis. IHC analysis of 71 GBM patients and 15 normal brain samples resulted in 26% staining in GBM patients and 71% staining in normal tissue, thus conflicting with the bioinformatic analysis. However, the authors proposed that the underlying mechanism was due to the bifunctional role of TGF-B as either an activator or inhibitor of cell proliferation, but this has yet to be researched in GBM in relation to HOXD10. A study by Yachi et al. found that miR-23a promotes invasion of GBM via HOXD10-regulated EMT [189]. miR-23a expression was significantly correlated to Matrigel invasion capacity in all six GBM cell lines tested. Bioinformatic analysis concluded that HOX10 was a reliable target of miR-23a, and in vitro overexpression of miR23a resulted in post-transcriptional degradation of HOXD10. Kaplan–Meier analysis using PrognoScan indicated decreased survival in patients with low HOXD10 expression, therefore confirming the IHC results presented by Li et al. It has been shown that HOXD10 suppresses the invasive capacity of GBM via uPAR, MMP14, and RhoC inhibition [190]. Thus, miR23a overexpression caused uPAR and RhoC expression, as well as the expression of the EMT markers Snail, Slug, MMP2, MMP9, and MMP14, with E-cadherin having decreased expression. However, HOXD10 overexpression reversed the alterations induced by miR-23a overexpression, therefore indicating HOXD10’s role as a tumor suppressor in GBM and a regulatory axis between miR-23a and HOXD10. The scratch wound-healing assay and Matrigel invasion were used to measure migration and invasive capacity in vitro. miR-23a overexpression significantly promoted invasion but not wound-healing capacity. HOXD10 overexpression reversed the invasion induced by miR-23a while also altering the morphology of the GBM cells from spindle shaped to flat with an enlarged cytoplasmic area, thus resulting in the reduction of invasion. Overall, these data implicate HOXD10 as a tumor suppressor in GBM and suggest that the miR-23a–HOXD10 regulatory axis plays a key role in the regulation of EMT in this cancer type.

### 12.3. Role of HOXA10 in Glioblastoma

He et al. researched the role of HOXA5 in GBM patient tissue and glioblastoma stem cells (GSCs) [191]. HOXA5 protein expression was analyzed in GBM tissue sections using IHC and fluorescence in situ hybridization (FISH). HOXA5 expression was significantly upregulated compared to low-grade glioma (LGG) and paired normal brain tissue in both IHC and FISH analyses. Bioinformatic analysis of TCGA patient datasets revealed that increased HOXA5 is associated with a significant decrease in overall survival in both GBM and LGG patients. HOXA5 mRNA expression was also upregulated, as indicated by the TCGA dataset and RNA-seq analysis of CD133+ GSC cell lines. GSEA and immunofluorescence staining revealed a positive correlation between HOXA5 expression and GSC markers in GBM samples, thus indicating that HOXA5 is associated with the GSC signature. As GSCs are required for GBM tumor initiation and the main cause of tumor recurrence [192,193], HOXA5 enrichment may be a powerful diagnostic tool and a potential therapeutic target. HOXA5’s role in GSC maintenance was further investigated. HOXA5 was silenced using shRNAs. HOXA5 in GSCs has a reduction of SOX2 and CD133 expression, both important markers for GSC formation and propagation [194,195]. The ability of GSCs to form tumorspheres was significantly reduced with HOXA5 silencing, indicating the role of HOXA5 in GSC self-propagation. In vivo HOXA5 silencing reduced GSC tumor-derived growth, and mice bearing HOXA5-silenced GSC lines had prolonged overall survival rates, indicating the importance of HOXA5 in GSC proliferation and tumor development. Upregulation of HOXA5 in non-stem tumor cells (NSTCs) resulted in increased tumorsphere formation and size, confirming the role of HOXA5 in GSCs. HOXA5 silencing in GSCs resulted in a significant decrease in invasion in transwell invasion chambers, with MMP-2 and MMP-7, two markers of cellular invasion, having decreased expression. Bioinformatic analysis to determine genes with HOXA5 binding sites revealed PTPRZ1. PTPRZ1 has been previously implicated in GBM migration and GSC maintenance [196,197]. A positive correlation between HOXA5 and PTPRZ1 expression in GBM was established using a luciferase reporter and ChiP assays. Lastly, PTPRZ1 silencing reduced the self-renewal capacity, invasiveness, and marker expression of GSCs, thus indicating that GSC maintenance relies on PTPRZ1 transcriptional activation by HOXA5. Taken together, these data suggest that enhanced levels of HOXA5 are associated with GSC propagation and invasion, therefore indicating the potential role of HOXA5 as a diagnostic and/or therapeutic target in GBM.

## 13. Conclusions

This narrative review discussed the clinical relevance of the function of HOX genes as diagnostic markers, therapeutic biomarkers, and/or prognostic indicators in a variety of solid and hematological malignancies (Table 1). Multiple studies, including those highlighted in this review, have shown that HOX gene dysfunction can orchestrate cancer progression and treatment resistance, specifically through epigenetic regulation or de novo dysregulation. We have seen that transcription factors, like the HOX gene family, exert significant influence on malignant transformation and potentiation. Specifically, HOX genes govern cancer advancement through direct regulation via transcriptional control or indirectly through methylation of numerous cancer-related genes. The vast breadth of HOX downstream targets is still widely unknown. Recent studies have shown that dysregulated HOX gene expression leads to aberrant regulation of genes related to proliferation, metastasis, angiogenesis, apoptosis, and chemotherapeutic resistance. HOX genes may function as oncodrivers or tumor suppressors depending on the cancer type and the form of dysregulation. HOX genes have unique expression signatures in different cancer types, thus acting as promising biomarkers for the development of diagnostic assays, enabling more precise stratification of patients and tailored therapeutic approaches. There is a crucial need to understand HOX downstream targets, as identification, may lead to advances in cancer treatment and positive patient outcomes. Additionally, targeted therapies that modulate HOX activity are being explored, as re-establishing normal HOX function could inhibit tumor growth. For example, the HOX/PBX dimerization is attributed to the carcinogenic phenotype of multiple malignancies [198]. HXR9, an 18 amino acid peptide inhibitor of this interaction, has been shown to reduce cell proliferation, tumor growth, and induce apoptosis, originally in melanoma cell lines [199]. HXR9 has been shown to be effective in a range of cancer cell types, including esophageal squamous cell carcinoma [200], glioblastoma [201], prostate cancer [202], oral squamous cell carcinoma [203], malignant mesothelioma [204], breast cancer [205], and ovarian cancer [206]. Furthermore, there has been some research on the differentiation inducer retinoic acid (RA) and the expression of HOX genes. RA has been shown to interact with HOX genes to drive differentiation during embryogenesis [207], modulate hematopoietic development [208], regulate cell proliferation, apoptosis, and metastasis [209,210], and maintain cellular identity and differentiation [211,212]. Overall, HOX genes represent a valuable frontier in cancer research, with the potential to improve both diagnosis and treatment strategies.

## Figures and Tables

**Table 1:** Reviewed HOX genes.

Gene Name	Cancer Type	Sample Type	Dysregulation Pattern	Clinical Relevance
HOXA1	Melanoma	Tissue	Overexpression	Prognostic indicator
	Lung cancer			Diagnostic marker/therapeutic biomarker
HOXA5	Chronic myeloid leukemia	Bone marrow	Hypermethylation	Prognostic indicator
	Gastric cancer	Tissue	Decreased expression	
	Colon cancer			
	Glioblastoma		Overexpression	
HOXA9	Papillary thyroid cancer	Tissue	Decreased Expression	Prognostic indicator
HOXA10	Acute myeloid leukemia	Bone marrow	Overexpression	Prognostic indicator/therapeutic biomarker
	Gastric cancer	Tissue		Prognostic indicator
HOXA13	Kidney renal clear cell carcinoma	Tissue	Overexpression	Prognostic indicator
	Non-small-cell lung cancer			
HOXB3	Lung adenocarcinoma	Tissue	Overexpression	Prognostic indicator
HOXB5	Acute myeloid leukemia	Bone marrow	Overexpression	Prognostic indicator
HOXB7	Multiple myeloma	Tissue	Overexpression	Prognostic indicator/therapeutic biomarker
HOXB9	Gastric cancer	Tissue	Overexpression	Prognostic indicator/therapeutic biomarker
HOXB13	Ovarian cancer	Tissue	Overexpression	Diagnostic marker/prognostic indicator
HOXC6	Multiple myeloma	Peripheral blood	Overexpression	Prognostic indicator/therapeutic biomarker
	Glioblastoma	Tissue	Decreased expression	Prognostic indicator
HOXC8	Breast cancer	Tissue	Decreased expression/methylation	Diagnostic marker
HOXC9	Gastric cancer	Tissue	Overexpression	Prognostic indicator
HOXC10	Melanoma	Tissue	Overexpression	Diagnostic marker
HOXC11	Kidney renal clear cell carcinoma	Tissue	Overexpression	Prognostic indicator
HOXC13	Melanoma	Tissue	Overexpression	Biomarker/prognostic indicator
HOXD1	Renal cancer	Tissue	Overexpression	Prognostic indicator/therapeutic biomarker
HOXD4	Ovarian cancer	Tissue	Overexpression	Prognostic indicator
HOXD9	Anaplastic thyroid cancer	Tissue	Overexpression	Diagnostic marker/prognostic indicator
HOXD10	Papillary thyroid cancer	Tissue	Decreased expression	Prognostic indicator
	Melanoma		Overexpression	Diagnostic marker
	Glioblastoma		Overexpression	Prognostic indicator/therapeutic and diagnostic marker
